# A prospective, randomized study of Toremifene vs. tamoxifen for the treatment of premenopausal breast cancer: safety and genital symptom analysis

**DOI:** 10.1186/s12885-020-07156-x

**Published:** 2020-07-16

**Authors:** Jin Hong, Jiahui Huang, Lili Shen, Siji Zhu, Weiqi Gao, Jiayi Wu, Ou Huang, Jianrong He, Li Zhu, Weiguo Chen, Yafen Li, Xiaosong Chen, Kunwei Shen

**Affiliations:** Department of General Surgery, Comprehensive Breast Health Center, Ruijin Hospital, Shanghai Jiao Tong University School of Medicine, 197 Ruijin Second Road, Shanghai, 200025 China

**Keywords:** Toremifene, Tamoxifen, Breast cancer, Premenopausal patients, Safety, Quality of life

## Abstract

**Background:**

Toremifene (TOR) is a selective oestrogen receptor modulator (SERM) and has comparable efficacy to that of tamoxifen (TAM) in breast cancer patients. Herein, we compared the safety of TOR to that of TAM in the adjuvant treatment of premenopausal breast cancer.

**Methods:**

This was a prospective randomized and open-label clinical study. Premenopausal patients with hormonal receptor (HR)-positive early breast cancer were randomly assigned (1:1) to receive TOR) or TAM treatment. The follow-up period was 1 year. The primary end point was the incidence of ovarian cysts, and secondary end points were the incidence of endometrial thickening, changes in female hormones, the incidence of fatty liver, changes in the modified Kupperman index (mKMI) and changes in quality of life.

**Results:**

There were 92 patients in the final analysis. The incidences of ovarian cysts were 42.6% in the TOR group and 51.1% in the TAM group (*p* = 0.441). Forty-one patients (87.2%) in the TOR group and 36 patients (80.0%) in the TAM group experienced endometrial thickening (*p* = 0.348). The proportions of patients with fatty liver were 31.9% in the TOR group and 26.7% in the TAM group (*p* = 0.581). No significant differences in the mKMI or quality of life were observed between the two groups.

**Conclusions:**

TOR and TAM have similar side effects on the female genital system and quality of life in premenopausal early breast cancer patients.

**Trial registration:**

ClinicalTrials.gov NCT02344940. Registered 26 January 2015 (retrospectively registered).

## Background

Endocrine therapy is a primary systemic therapy for hormonal receptor (HR)-positive breast cancer. Tamoxifen (TAM) is a selective oestrogen receptor modulator (SERM) that competitively inhibits oestrogen binding to oestrogen receptor (ER) and is effective in both pre- and postmenopausal women [1]. A meta-analysis showed that compared with no endocrine therapy, adjuvant TAM for 5 years reduced the 5-year breast cancer recurrence rate by approximately 50% in HR-positive breast cancer [2]. A longer duration of TAM has also been suggested for specific premenopausal breast cancer patients [3, 4].

However, TAM often causes a range of adverse events, such as hot flashes, endometrial hyperplasia or uterine cancer, ovarian cyst formation and thromboembolic disease [1, 5, 6]. Hot flashes are the most common side effect, affecting approximately 42.9% of patients taking TAM [7]. Ovarian cysts are diagnosed in 17–19% of patients treated with TAM, and in premenopausal women, the proportion of ovarian cysts varies from 30 to 49% [6, 8]. There is little risk of endometrial cancer in patients younger than 54 years [2]. Additionally, TAM causes other adverse events, such as fatty liver and lipid changes [9, 10].

Toremifene (TOR) is another SERM option for the treatment of HR-positive breast cancer and differs from TAM in structure by only one chlorine atom [11]. In postmenopausal patients, TOR has been verified to have similar efficacy to that of tamoxifen as an adjuvant treatment and for metastatic disease [11–14]. In contrast to TAM, which is metabolized by cytochrome P450 enzymes, TOR is not a prodrug and has better efficacy in breast cancer patients with the CYP2D6*10 T/T genotype [15]. Data on the efficacy of TOR in premenopausal patients are limited. A retrospective study revealed that TOR had a 5-year overall survival rate that was similar to that of TAM and an even better recurrence-free survival rate than that of TAM [16]. To date, there are no data regarding comparisons of the side effects of TOR versus TAM in premenopausal breast cancer patients. Herein, we carried out a prospective clinical study to evaluate the safety of TOR versus TAM in premenopausal patients with early breast cancer.

## Methods

### Study design and treatment

This was a prospective, single-centre, randomized, controlled, and open-label clinical study. All participants were from Ruijin Hospital, Shanghai Jiao Tong University, School of Medicine. Premenopausal patients with HR-positive early breast cancer who were scheduled to receive SERMs as adjuvant endocrine therapy after discussion by the multidisciplinary team (MDT) were recruited. A block randomization method with a block size of 6 was used to achieve balance between treatment groups by the investigator. There was no stratification for the study. Patients were enrolled by doctors in our centre. Sequentially numbered opaque sealed envelopes were used for allocation concealment and managed by the oncology nurse specialist.

After surgery, chemotherapy, and radiotherapy, patients were randomly assigned at a 1:1 ratio to the TOR group and the TAM group. Patients in the TOR group received TOR citrate tablets (60 mg/day), and patients in the TAM group received TAM citrate tablets (20 mg/day). All patients were followed up every 3 months in the first year from endocrine therapy initiation.

### Eligibility criteria

Patients were included if they met the following criteria: were premenopausal women; had histologically confirmed HR-positive breast cancer; underwent standard surgery for breast cancer; had completed other adjuvant therapy, such as chemotherapy and radiotherapy; had leukocyte counts ≥3.0 × 10^9^/L and platelet counts ≥75 × 10^9^/L; had serum alanine aminotransferase (ALT) or aspartate aminotransferase (AST) levels that were ≤ 2.5 times the upper limit of normal range (ULN); had serum creatinine levels less than the ULN; and had an Eastern Cooperative Oncology Group (ECOG) performance score of 0–2. The exclusion criteria were as follows: HR negative; previous neoadjuvant or adjuvant endocrine therapy administration; metastatic malignancies; family history of endometrial cancer or other gynaecologic malignant tumours; ovarian cysts (largest diameter ≥ 2 mm) by transvaginal ultrasound (TVU); hysterectomy or ovariectomy surgery; any complication that increased sex hormone secretion, such as thymic cancer, ovarian tumour or pituitary adenoma; any complication that decreased sex hormone secretion, such as hyperthyroidism, hypothyroidism, severe malnutrition, liver cirrhosis, sex hormone synthetase deficiency, Turner’s syndrome, intracranial tumour, or a pituitary condition; a severe non-malignant comorbidity that could influence long-term follow-up; severe cardiac dysfunction; severe hepatic dysfunction, Child-Pugh C; or a known severe hypersensitivity to any drug in this study.

### Clinicopathological information

The patients’ clinical information was collected from the case report forms of the study. Medical history data included age, menstrual status, ECOG score, past medical history, biochemical parameters and the parameters of routine blood tests. Other treatment information included chemotherapy, radiotherapy, and targeted therapy. Pathological results were reported by two different pathologists independently and included pathological type, tumour size, histological grade, lymph node involvement, ER expression, progesterone receptor (PR) expression, CerbB-2 status and the result of the fluorescence in situ hybridization (FISH) test. ER or PR positivity was defined as nuclear staining in more than 1% of tumour cells. Tumours with a CerbB-2 3+ status in the immunohistochemistry assay and/or human epidermal growth factor receptor-2 (HER-2) gene overexpression confirmed by FISH were defined as HER-2 positive.

Serum oestradiol (E2), follicle-stimulating hormone (FSH) and luteinizing hormone (LH) were measured at baseline and every 3 months after randomization by the gynecological clinical Lab in our hospital. Hormone levels were analyzed using commercially available kits from the Unicel DXI 800 Access immunoassay system (Beck-man Coulter).

### Study end points

The primary end point of the study was the incidence of ovarian cysts, which were defined as pure liquid-filled structures that were equal to or greater than 2.0 cm at their largest diameters by TVU. The secondary end points were as follows: the incidence of endometrial thickening (endometrium ≥8.0 mm measured by TVU), changes in female hormones (E2, FSH and LH), the incidence of fatty liver (detected by abdominal ultrasound according to the criteria of the American Association for the Study of Liver Disease [17]), changes in the modified Kupperman Menopausal index (mKMI) and changes in quality of life.

### Assessment of menopausal symptoms

The mKMI was used to evaluate menopausal symptoms [18]. The mKMI consists of 13 items: hot flashes/sweats, palpitation, vertigo, headache, paraesthesia, formication, arthralgia, myalgia, fatigue, nervousness, melancholia, urinary infections and sexual complaints. Each item was divided into four grades (0–3 points) according to severity: 0, no symptoms; 1, mild symptoms; 2, moderate symptoms; and 3, severe symptoms. The total scores ranged from 0 to 63, and score ranges of 0–6, 7–15, 16–30 and > 30 represented the degrees of severity, namely, none, mild, moderate and severe, respectively [18]. Patients were asked to complete the mKMI questionnaire at baseline and then every 3 months.

### Quality of life assessment

Quality of life was assessed using the European Organization for Research and Treatment of Cancer (EORTC) QLQ-C30 (version 3.0), which consists of 30 questions addressing five functional scales (cognitive, emotional, physical, social, and role), nine symptom scales (appetite loss, constipation, diarrhoea, dyspnoea, fatigue, financial difficulties, insomnia, nausea and vomiting, and pain) and one global health status scale [19]. The EORTC-QLQ-C30 questionnaires were completed at baseline and then every 6 months.

### Statistical analysis

The study was designed to have a power of 90% to detect an absolute reduction of 20% for the incidence of ovarian cysts in patients treated with toremifene compared to patients treated with tamoxifen (15% vs 35%), at a one-sided significance level of 0.05. Taking a withdrawal rate of 15% into consideration, the target enrolment was 52 eligible patients for each group based on the Simon 2-stage design.

Categorical variables between two groups are presented as frequencies and percentages and were compared using chi-square tests (the 2-sided Pearson) or Fisher’s exact test. Continuous data are presented as the mean ± standard deviation (SD) or mean ± standard error (SE) and were compared using a nonparametric test (Mann-Whitney U). The analysis was performed using SPSS (version 22.0) software (IBM Corporation, Armonk, NY, USA), and figures were generated by GraphPad Prism (version 5) (GraphPad Software, San Diego, CA, USA). A *p* value < 0.05 was considered to indicate statistical significance.

## Results

### Study population

**Fig. 1:**
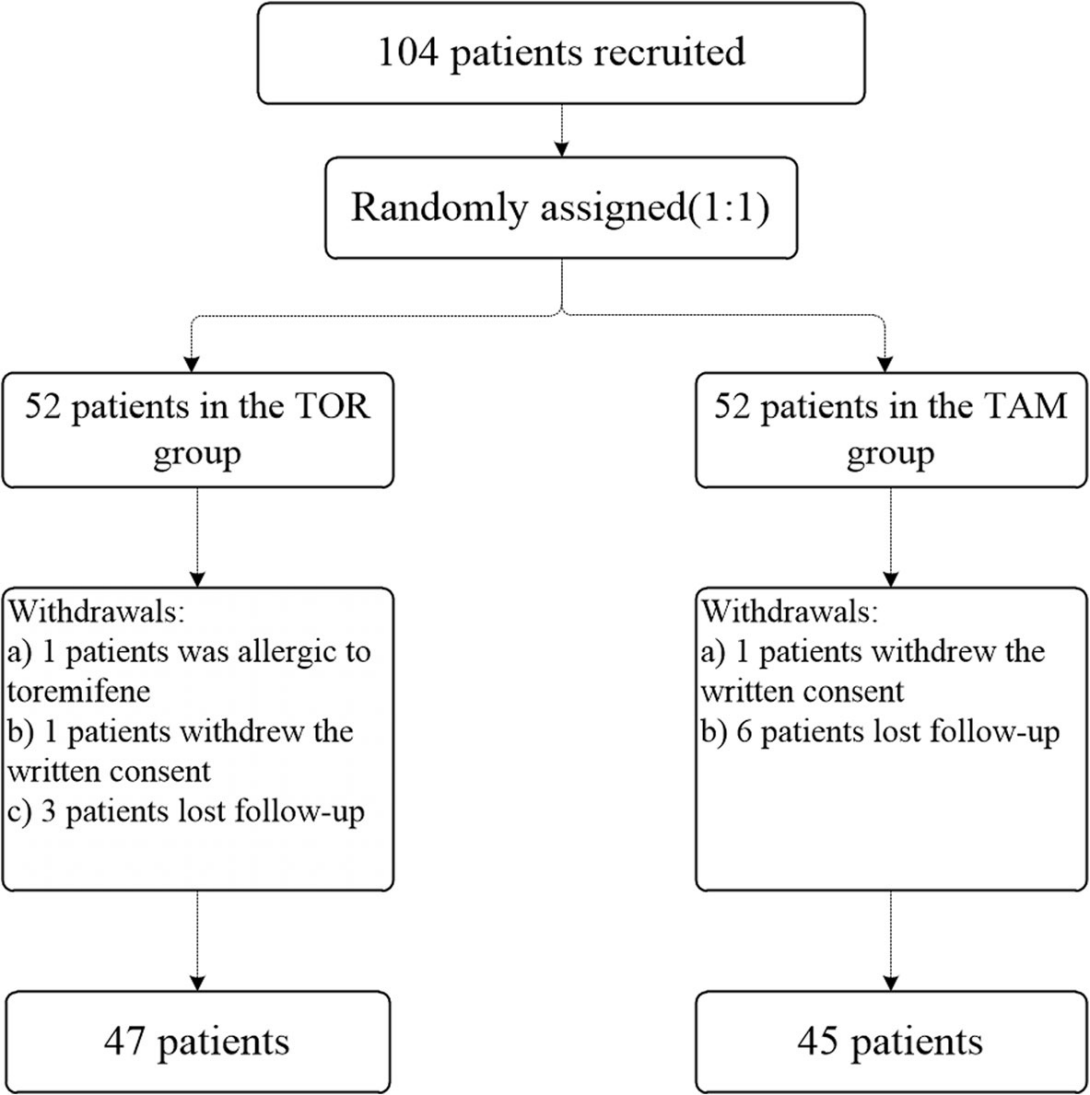
Flow Chart of Patient’s Enrollment

From December 2014 to June 2017, 104 patients were recruited and randomized to receive either toremifene (*N* = 52) or tamoxifen (N = 52) treatment. Twelve patients did not receive the study drugs: one suffered from a severe rash in the toremifene group, two received non-study drugs due to personal reasons, and nine were lost to follow-up (Fig. 1). Finally, a total of 92 patients were collected in the final analysis.

The patients’ clinicopathological characteristics and adjuvant treatments were well balanced between the two groups (Table 1). The median ages were 45 years in the TOR group and 44 years in the TAM group. Regarding the tumours of these patients, 78.3% were invasive ductal carcinomas, and 7 were ductal carcinomas in situ. Eighty-one patients had stage I or II breast cancer. Immunohistochemistry showed that 86 (93.5%) tumours were more than 50% ER positive, that 69 (75%) tumours were more than 20% PR positive, and that only 6 patients were HER-2 positive. Regarding adjuvant treatment, 33 patients received chemotherapy, 44 patients received radiotherapy, and 4 patients received trastuzumab treatment. All patients were menstruating before surgery, 6 patients in the TOR group and 10 patients in the TAM group had chemotherapy induced amenorrhea.

**Table 1:** Baseline Patient characteristics and treatment

Characteristic	Overall, n(%)	Groups		*p*-value
		TOR	TAM	
Age				0.053
< 40	12(13.0)	3	9	
≥ 40	80(87.0)	44	36	
BMI				0.880
< 24	73(79.3)	37	36	
≥ 24	19(20.7)	10	9	
Surgery				0.273
Mastectomy	54(58.7)	25	29	
BCS	38(41.3)	22	16	
Pathology				0.890
IDC	72(78.3)	37	35	
DCIS	7(7.6)	4	3	
Others	13(14.1)	6	7	
Tumors				0.717
≤ 2.0 cm	67(72.8)	35	32	
> 2.0 cm	25(27.2)	12	13	
Nodes status				0.830
Negative	79(85.9)	40	39	
Positive	13(14.1)	7	6	
Grade				0.655
I	15(16.3)	9	6	
II	47(51.1)	25	22	
III	11(12.0)	4	7	
NA	19(20.7)	9	10	
ER				0.430
≥ 50%	86(93.5)	43	43	
< 50%	6(6.5)	4	2	
PR				0.399
≥ 20%	69(75)	37	32	
< 20%	23(25)	10	13	
HER-2				0.430
positive	6(6.5)	4	2	
negative	86(93.5)	43	43	
Subtype				0.164
Luminal A like	40(43.5)	24	16	
Luminal B HER2 negative	46(50.0)	19	27	
Luminal B HER2 positive	6(6.5)	4	2	
Ki67				0.554
< 14%	58(63.0)	31	27	
≥ 14%	34(37.0)	16	18	
Chemotherapy				0.709
Yes	33(35.9)	16	17	
No	59(64.1)	31	28	
Radiotherapy				0.828
Yes	44(47.8)	23	21	
No	48(52.2)	24	24	
Trastuzumab				0.328
Yes	4(4.3)	3	1	
No	88(95.7)	44	44	

Abbreviations: *BMI* Body Mass Index, *IDC* Invasive ductal carcinoma, *DCIS* Ductal carcinoma in situ, *NA* Not available, *BCS* Breast conserving surgery, *ER* Estrogen receptor, *PR* Progestrone receptor, *TAM* Tamoxifen, *TOR* Toremifene

*p < 0.05 was considered statistically significant

### Incidence of ovarian cyst formation

**Fig. 2:**
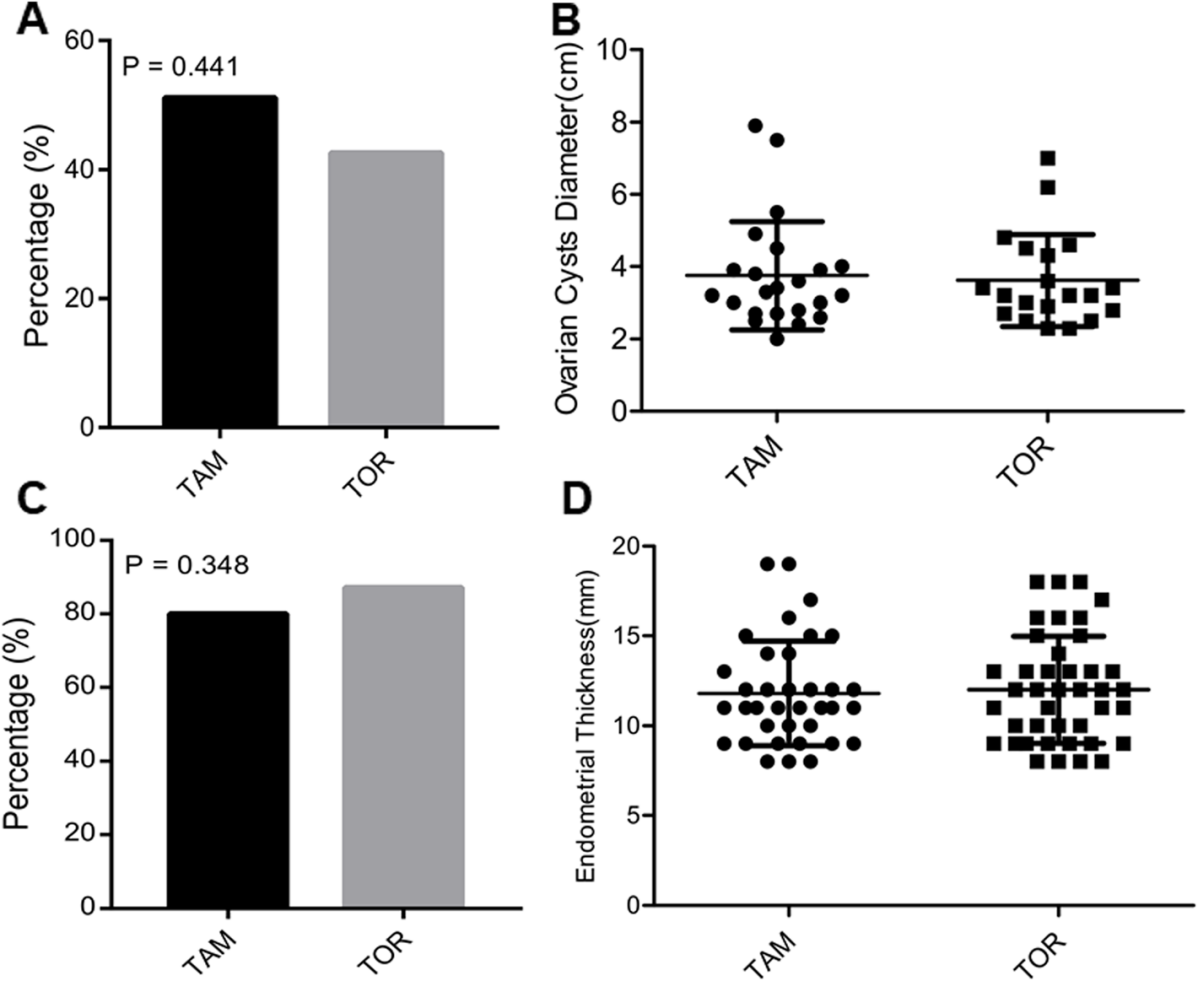
Incidences of ovarian cysts and endometrial thickening in premenopausal women treated with tamoxifen or toremifene. **a** Percentage of ovarian cysts (largest diameter ≥ 2 cm); **b** Mean values of ovarian cysts diameters in two groups. **c** Percentage of endometrial thickening (endometrial thickness ≥ 8 mm) in two groups; **d** Mean values of endometrial thickness for patients with endometrial thickening in two groups. Abbreviation: *TAM* tamoxifen, *TOR* toremifene

After 1 year of follow-up every 3 months, 20 patients (42.6%) in the TOR group and 23 patients (51.1%) in the TAM group had ovarian cysts (largest diameter ≥ 2.0 cm) detected by TVU (Fig. 2a). The mean values of the ovarian cyst diameters were 3.62 ± 1.27 cm in the TOR group and 3.75 ± 1.50 cm in the TAM group (Fig. 2b, *p* = 0.789). There was no significant difference in the incidence of ovarian cysts between the two groups (OR = 1.411, 95% CI = 0.620–3.211, *p* = 0.441).

The percentages of ovarian cysts (largest diameter ≥ 3.0 cm) were 27.7% in the TOR group and 37.8% in the TAM group, and there was no significant difference between the two groups (OR = 1.588, 95% CI = 0.660–3.822, *p* = 0.301).

### Incidence of endometrial thickening

The incidences of endometrial thickening in premenopausal patients treated with toremifene and tamoxifen are shown in Fig. 2c. The mean endometrial thicknesses were 12.00 ± 2.98 mm in the toremifene group and 11.80 ± 2.91 mm in the tamoxifen group (Fig. 2d, *p* = 0.723). Forty-one patients (87.2%) in the TOR group and 36 patients (80.0%) in the TAM group had endometrial thickening (endometrium ≥8.0 mm) during the one-year follow-up period. No significant difference in the incidence of endometrial thickening was observed between the two groups (OR = 0.585, 95% CI = 0.190–1.805, *p* = 0.348).

### Changes in plasma FSH, LH, and E2 concentrations

**Fig. 3:**
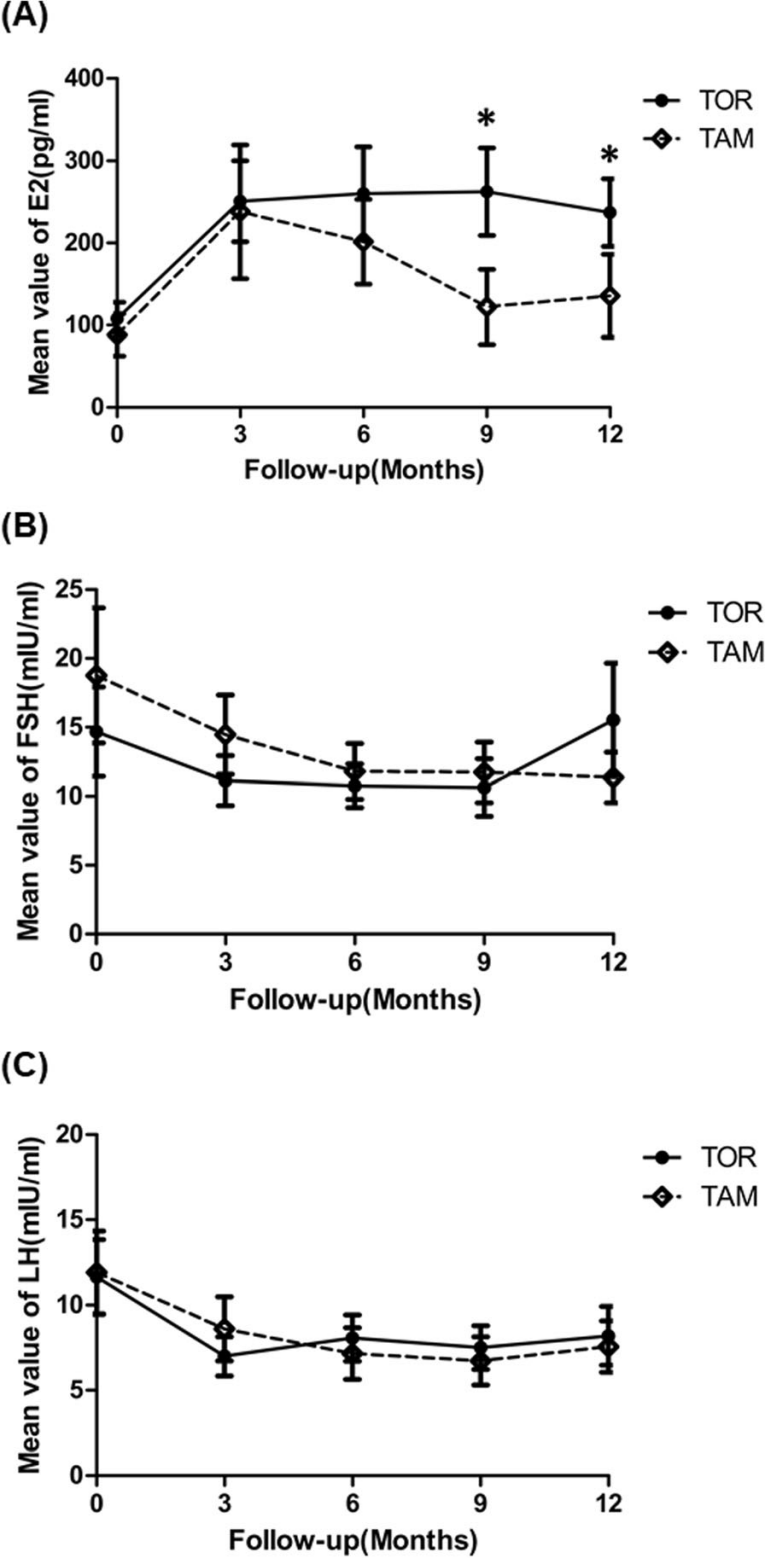
Mean values (±SE) of serum E2(**a**), FSH(**b**) and LH(**c**) and in patients treated with toremifene and tamoxifen at each follow-up. * *p* < 0.05. Abbreviation: *TAM* tamoxifen, *TOR* toremifene, *E2* estradiol, *FSH* follicle stimulating hormone, *LH* luteinizing hormone

Among 92 patients, 23 in the TOR group and 17 in the TAM group had complete serum E2, FSH, and LH data at each follow-up. The mean values for E2, FSH and LH at each follow-up in the two groups are presented in supplementary Table 1. Figure 3a shows the mean E2 value. At baseline, the mean E2 values were 102.96 pg/L in the TOR group and 88.24 pg/L in the TAM group. This concentration increased to 262.39 pg/L at the 9th month of toremifene treatment. In the TAM group, the mean E2 level increased to 238.12 pg/L at the 3rd month and decreased thereafter. The mean E2 values at the 9th month (*p* = 0.042) and 12th month (*p* = 0.018) were significantly higher in the TOR group than in the TAM group. The mean values for FSH and LH were in the normal range at each follow-up exam. There were no significant differences between the two groups.

### Incidence of fatty liver

Fifteen of 47 patients in the TOR group and 12 of 45 patients in the TAM group developed fatty liver during one year of endocrine therapy. There was no significant difference between the two groups (31.9% vs 26.7%, OR = 0.776, 95% CI = 0.315–1.911, *p* = 0.581).

### Assessment of menopausal symptoms and quality of life

The numbers of patients who completed the mKMI questionnaire and the mean mKMI scores of the two groups at baseline and for every 3 months of follow-up are listed in Table 2. There were no significant differences in the mean mKMI scores between the TOR group and the TAM group at each follow-up. The highest mean mKMI score was 13.00 at the 6th month in the TOR group and 13.03 at the 9th month in the TAM group.

**Table 2:** Mean values of mKMI in two groups at each follow-up

Time	TOR		TAM		*p-*value
	N	Mean (SD)	N	Mean (SD)	
Baseline	46	10.91(7.34)	45	9.53(6.40)	0.530
3-month follow-up	45	11.78(8.31)	37	13.00(6.97)	0.285
6-month follow-up	44	13.00(8.92)	39	12.49(6.31)	0.949
9-month follow-up	43	10.79(8.33)	35	13.03(7.01)	0.165
12-month follow-up	40	12.70(8.81)	34	11.59(6.08)	0.761

Abbreviations: *mKMI* Modified Kupperman Menopausal Index, *SD* Standard deviation, *TAM* Tamoxifen, *TOR* Toremifene

*p < 0.05 was considered statistically significant

The EORTC-QLQ-C30 questionnaire was used to assess quality of life. The mean scores of the functional scales and symptom scales were compared between the TOR group and TAM group. There were no significant differences in any scale between the two groups throughout follow-up (Table 3). The mean scores of all functional scales increased gradually except for the mean score of cognitive functioning, which decreased from 88.62 at baseline to 84.90 at 12 months in the TAM group. The mean score of the appetite loss scale in the TOR group was slightly higher than that in the TAM group, with marginal significance (14.73 vs 5.56, *p* = 0.051), at the 6th month of follow-up.

**Table 3:** Quality of life assessment based on EORTC-QLQ-C30

EORTC-QLQ-C30 scales	Baseline		*p-*value	6-months follow-up		*p-*value	12-months follow-up		*p-*value
	Toremifene	Tamoxifen		Toremifene	Tamoxifen		Toremifene	Tamoxifen	
	Mean (SD)	Mean (SD)		Mean (SD)	Mean (SD)		Mean (SD)	Mean (SD)	
Functional scales									
Physical functioning	86.05(12.75)	83.58(11.56)	0.276	88.68(12.13)	88.33(13.46)	0.968	90.10(11.36)	91.25(9.79)	0.849
Role functioning	73.64(21.59)	79.27(20.68)	0.211	84.50(20.70)	81.48(21.37)	0.427	89.05(17.59)	86.46(16.09)	0.361
Emotional functioning	70.93(20.92)	81.71(18.75)	0.017	76.16(22.76)	80.56(13.21)	0.667	79.76(19.83)	82.81(13.04)	0.798
Cognitive functioning	82.56(18.17)	88.62(12.04)	0.138	83.72(16.46)	85.19(13.08)	0.932	84.29(21.37)	84.90(18.14)	0.825
Social functioning	72.48(22.97)	78.46(20.50)	0.291	82.17(20.70)	85.64(19.17)	0.469	89.52(15.17)	87.50(16.40)	0.543
Symptom scales									
Fatigue	31.52(19.39)	25.20(14.70)	0.080	25.06(19.86)	25.00(14.64)	0.763	23.17(24.16)	18.75(16.80)	0.664
Nausea/vomitting	3.10(11.65)	5.28(13.14)	0.198	3.10(8.34)	1.39(6.14)	0.229	1.90(6.73)	2.08(7.02)	0.909
Pain	22.87(22.13)	16.67(13.94)	0.292	17.44(18.17)	12.5(14.57)	0.215	12.38(13.61)	16.15(16.11)	0.368
Dyspnoea	18.60(28.45)	12.19(17.89)	0.211	13.18(20.75)	12.04(16.24)	0.924	15.24(21.91)	15.63(20.71)	0.850
Insomnia	13.17(25.34)	28.46(26.42)	0.353	30.23(34.74)	28.70(21.31)	0.620	30.48(33.70)	23.96(25.73)	0.571
Appetite loss	18.60(28.45)	9.76(17.07)	0.200	14.73(22.19)	5.56(12.60)	0.051	11.43(19.71)	5.21(12.30)	0.180
Constipation	13.18(25.34)	12.20(19.37)	0.655	16.28(28.52)	6.49(17.49)	0.080	19.82(33.75)	13.54(20.50)	0.845
Diarrhoea	6.20(13.12)	7.32(19.02)	0.898	3.88(10.81)	5.56(14.91)	0.733	4.50(16.03)	2.08(8.20)	0.728

*p < 0.05 was considered statistically significant

## Discussion

Our prospective study found that in HR-positive premenopausal breast cancer patients, the incidence of ovarian cysts was similar between the TOR and TAM groups. Additionally, other side effects, such as endometrial thickening, menopausal symptoms, fatty liver, and quality of life, were all comparable between the two groups.

TAM is the dominant endocrine therapy for premenopausal breast cancer patients; however, approximately 42% of patients discontinue treatment within the first 2 years for different reasons [20]. In addition to its anti-oestrogenic effects, TAM has a mild oestrogenic effect that depends on the end organ, endogenous oestrogen levels and dose [8]. Gynaecologic symptoms and side effects on the ovary and uterus are the most common adverse events in patients receiving SERMs [5]. TOR is another nonsteroidal triphenylethylene selective ER modulator and has similar efficacy in patients with breast cancer [14, 21]. A previous meta-analysis showed that TOR and TAM have similar severe adverse events between peri- or postmenopausal patients but that TOR may cause less vaginal bleeding, fewer headaches and fewer thromboembolic events [14, 21]. However, there is no prospective clinical study that evaluates and compares the adverse effects of TOR and TAM in premenopausal women.

Ovarian cysts are common in premenopausal women treated with TAM and are associated with higher serum E2, younger age and the absence of high-dose chemotherapy [22]. Between different studies, the incidence of ovarian cysts in premenopausal patients ranges from 17 to 49%, and these rates are higher than that in postmenopausal women [8, 22]. There are few reports on ovarian cysts in patients treated with TOR. Our results revealed that the incidence of ovarian cysts detected by TVU was similar between the TOR and TAM patient groups, though slightly higher numerically in the TAM group. We observed that the incidence of ovarian cysts was very high in premenopausal patients treated with SERMs: 42.6 and 51.5% in the TOR and TAM groups, respectively.

Treatment with TAM can increase plasma E2 concentrations by interfering with normal negative pituitary feedback mechanisms [23, 24]. A previous study revealed that circulating levels of FSH and LH remained in the normal range in premenopausal patients who received TAM, while the level of E2 was elevated one- to three-fold [25]. Our study had the same results: plasma E2 increased significantly with TOR or TAM treatment, and the levels of FSH and LH remained in the normal range. However, we found that the mean E2 values in the TAM group decreased after the second follow-up examination and were lower than those in the TOR group.

Because of its oestrogen-like effects on the uterus, TAM also triggers endometrial proliferation [26]. In previous studies, endometrial thickness was significantly higher in postmenopausal patients treated with TAM than in control subjects [27, 28]. Severe gynaecologic adverse events, such as endometrial polyps, hyperplasia and endometrial cancer, induced by TAM were increased by 2–4-fold compared to no TAM treatment [29]. Long follow-up studies have shown that 5 years of adjuvant TAM brings about a 2–3% risk of endometrial cancer over 15 years; however, there is little risk of endometrial cancer in premenopausal women [4]. All patients in our study were premenopausal women, and the follow-up time was only 1 year; no patient underwent endometrial biopsy due to endometrial thickening, and no endometrial cancer was found. However, the proportions of endometrial thickening in the TAM and TOR groups were approximately 80 and 87.2%, respectively, although there was no significant difference between the two groups. The measurement of endometrial thickness was difficult as the menstrual pattern changes in premenopausal patients taking TAM [23, 30]. For patients with a regular menstrual cycle, TVU was carried out 1 week after menses every 3 months. For patients with irregular menstrual cycles, oligomenorrhea or amenorrhea, TVU was carried out every 3 months routinely. At baseline, 36 patients (39.1%) already had endometrial thickening. A previous study of patients in Japan showed that the median endometrial thickness was 8.6 mm [31]. As the cut-off of endometrial thickness was 8 mm in our study, we observed high rates of endometrial thickening.

As TAM treatment also influences ovarian function, approximately two-thirds of patients develop oligomenorrhea or amenorrhea, which leads to side effects such as hot flashes [23]. We used the mKMI to evaluate menopausal symptoms. At baseline, the mean mKMI scores were 10.91 in the TOR group and 9.53 in the TAM group. During follow-up, the mean scores of the two groups increased slightly compared with the baseline scores. No significant differences between the two groups were observed at any of the follow-up times. In terms of the severity grade, both the patients treated with TOR and those treated with TAM had mild menopausal symptoms.

Non-alcoholic fatty liver disease (NAFLD) is another common adverse event caused by TAM, and may be related to increasing serum triglycerides, inhibition of mitochondrial β-oxidation of fatty acids and suppression of oestrogen synthesis [10]. Previous studies revealed that the NAFLD rates in patients taking TAM were approximately 46 to 48% [10, 32]. In patients treated with TOR, the incidence of NAFLD was only 7.7%, as reported by a study from Japan [33]. Our results showed that in the first year of endocrine therapy, 31.9% of patients in the TOR group and 26.7% of patients in the TAM group had NAFLD, and these incidences were slightly lower than those of previous reports. However, we did not observe the superiority of TOR over TAM. In addition, some studies revealed that prevalence of NAFLD was ranging from 25 to 44% in China [34]. It was a confusing factor that we could not make the comparison simply.

Quality of life was evaluated by means of the EORTC-QLQ-C30 questionnaire, and there were no significant differences between the two groups at any of the follow-up times. We also observed an increasing tendency in the mean scores of the functional scales and a slight decreasing tendency in the mean scores of the symptom scales compared with the baseline, indicating that quality of life was improved with both TOR and TAM treatment. Although controversial, the results of other studies support this phenomenon, wherein quality of life at baseline is worse after surgery or chemotherapy [35, 36].

This study has some limitations. The current study is an open-label study, and block randomization may result in selection bias when the study groups are unmasked. Second, gynaecological side effects in patients are also influenced by other factors, such as chemotherapy and radiotherapy. Third, the follow-up time of this study was only 1 year and was too short for the detection of some adverse events, such as endometrial cancer. Forth, Sex hormones were analysed in less than half of the patients.

## Conclusions

In conclusion, our prospective study revealed that treatment with TOR or TAM results in similar side effects in terms of the female genital system and quality of life in premenopausal women with breast cancer. The incidence rates of ovarian cysts were similar between the TOR and TAM groups. Other side effects, such as endometrial thickening, menopausal symptoms and fatty liver, were comparable between the two groups. TOR is a safe alternative to TAM as an adjuvant treatment for HR-positive premenopausal breast cancer.

## Supplementary information

**Additional file 1 Table S1.** Mean values of FSH, LH and E2 in two groups at each follow-up.

## Data Availability

The datasets during and/or analysed during the current study are available from the corresponding author on reasonable request.

## Abbreviations

HR: Hormonal receptor
TOR: Toremifene
TAM: Tamoxifen
mKMI: Modified Kupperman Menopausal index
SERM: Selective estrogen receptor modulator
ER: Estrogen receptor
PR: Progesterone receptor
HER-2: Human epidermal growth factor receptor-2
ECOG: Eastern Cooperative Oncology Group
ALT: Alanine aminotransferase
AST: Aspartate aminotransferase
ULN: Upper limit of normal range
TVU: Transvaginal ultrasound
FISH: Fluorescence in situ hybridization
E2: Plasma estradiol
FSH: Follicle stimulating hormone
LH: Luteinizing hormone
EORTC: European Organization for Research and Treatment of Cancer
SD: Standard deviation
SE: Standard error
NAFLD: Non-alcoholic fatty liver disease
