# Supplementary material for: A prospective, randomized study of Toremifene vs. tamoxifen for the treatment of premenopausal breast cancer: safety and genital symptom analysis
Source: BMC Cancer. 2020 Jul 16;20:663. doi: 10.1186/s12885-020-07156-x (PMC7364473; doi:10.1186/s12885-020-07156-x)
Supplement: Supplementary file 1 — Additional file 1 Table S1. Mean values of FSH, LH and E2 in two groups at each follow-up. [file 12885_2020_7156_MOESM1_ESM.docx]

Supplementary Table1. Mean values of FSH, LH and E2 in two groups at each follow-up

| Follow-up | E2 | | | p-value | FSH | | | p-value | LH | | | p-value |
| --- | --- | --- | --- | --- | --- | --- | --- | --- | --- | --- | --- | --- |
|  | TOR |  | TAM |  | TOR |  | TAM |  | TOR |  | TAM |  |
|  | Mean (SE) |  | Mean (SE) |  | Mean (SE) |  | Mean (SE) |  | Mean (SE) |  | Mean (SE) |  |
| Baseline | 107.96(19.97)  250.83(49.20)  260.04(56.98)  262.39(53.30)  237.01(41.11) |  | 88.24(25.61)  238.12(81.48)  201.51(51.56)  122.12(45.69)  135.65(50.45) | 0.273 | 14.68(3.24)  11.13(1.81)  10.76(1.58)  10.64(2.08)  15.54(4.11) |  | 18.77(4.90)  14.47(2.88)  11.81(2.02)  11.73(2.19)  11.36(1.83) | 0.924 | 11.66(2.20)  7.00(1.15)  8.07(1.36)  7.51(1.28)  8.20(1.73) |  | 11.92(2.43)  8.61(1.88)  7.16(1.51)  6.73(1.42)  7.56(1.51) | 0.924 |
| 3 months |  |  |  | 0.477 |  |  |  | 0.381 |  |  |  | 0.702 |
| 6 months |  |  |  | 0.493 |  |  |  | 0.763 |  |  |  | 0.331 |
| 9 months |  |  |  | 0.042 |  |  |  | 0.547 |  |  |  | 0.547 |
| 12 months |  |  |  | 0.018 |  |  |  | 0.870 |  |  |  | 0.642 |

*FSH* follicle stimulating hormone, *LH* luteinizing hormone, *E2* estradio, *TAM* tamoxifen, *TOR* toremifene, *SE* Standard Error

*p<0.05 was considered statistically significant
